# Beyond Psoriasis Skin Clearance: Effects of Bimekizumab and Tildrakizumab on Systemic Inflammation and Immune-Nutrition Status

**DOI:** 10.3390/ph19101523

**Published:** 2026-09-25

**Authors:** Elena Campione, Fabio Artosi, Pamela Masci, Chiara Cattani, Giulia Marrone, Sara Lambiase, David Della-Morte, Luca Bianchi, Francesca Pacifici

**Affiliations:** 1 Dermatology Unit, Department of Systems Medicine, Tor Vergata University Hospital, University of Rome Tor Vergata, 00133 Rome, Italy; elena.campione@uniroma2.it (E.C.); fabio.artosi994@gmail.com (F.A.); mascipamela99@gmail.com (P.M.); chiara.cattani@gmail.com (C.C.); sara.lambiase@alice.it (S.L.); luca.bianchi@uniroma2.it (L.B.); 2 UOSD Nephrology and Dialysis, Policlinico Tor Vergata, University of Rome Tor Vergata, 00133 Rome, Italy; giulia.marrone@uniroma2.it; 3 Section of Food Chemistry, Clinical Nutrition and Pharmaceutical Science, Department of Biomedicine and Prevention, University of Rome Tor Vergata, Via Montpellier 1, 00133 Rome, Italy; david.dellamorte@uniroma2.it; 4 Department of Neurology, Evelyn F. McKnight Brain Institute, University of Miami Miller School of Medicine, 1120 NW 14th Street, Miami, FL 33136, USA; 5 Hepatology, Clinical Nutrition and Geriatrics Unit, Policlinico Tor Vergata (PTV) Hospital, University of Rome Tor Vergata, 00133 Rome, Italy; 6 Department for the Promotion of Human Sciences and Quality of Life, San Raffaele Roma Open University, 00166 Rome, Italy

**Keywords:** HALP score, CONUT score, PASI, DLQI

## Abstract

**Background/Objectives**: Psoriasis is a chronic immune-mediated inflammatory disease associated with a high systemic burden. Inhibition of the IL-23/IL-17 axis is the cornerstone of therapy, although the evaluation of immune-nutritional status in the assessment of psoriatic disease-associated comorbidities is an equally crucial topic. The aim of the study was to document the efficacy, safety and systemic impact of Bimekizumab and Tildrakizumab in patients with chronic plaque psoriasis by analyzing clinimetric indices, atherogenic risk, and immune-nutritional markers. **Methods**: This was a descriptive real-world clinical study, including 52 psoriasis patients treated with Bimekizumab or Tildrakizumab, who were followed for 52 weeks. In particular, we analyzed the clinimetric (evaluated by PASI and DLQI) and inflammatory (CRP, ESR, NLR, PLR, and LMR ratios; SII) indices, atherogenic risk (by evaluating the lipid profile), and immuno-nutritional markers (HALP score and CONUT score). Assessments were performed at baseline and after 52 weeks. **Results**: Both treatments led to a significant reduction in the PASI at week 52. The systemic inflammatory marker CRP decreased in both groups. A trend of reduction from baseline to week 52 was reported for both the NLR and PLR. Conversely, a trend of increase was reported for the LMR. The HALP index increased significantly in the Bimekizumab group but not in the Tildrakizumab group, while the CONUT score was significantly lower, suggesting an improvement in the immune-nutritional status. **Conclusion**: In conclusion, the integrated assessment of hematological ratios, inflammatory indices and immune-nutritional markers provides clinically useful information for a more complete and personalized management of psoriatic patients.

## 1. Introduction

Psoriasis is one of the most relevant immune-mediated inflammatory dermatological diseases [1,2,3,4], with a global prevalence of 4.4% [5]. Its pathogenesis is multifactorial, with genetics as a main contributor [1]. Psoriasis is associated with several comorbidities, including cardiovascular disease, diabetes, dyslipidemia and metabolic syndrome [6,7], which share a common underlying pro-inflammatory state. All this supports the hypothesis that psoriasis should be considered a systemic inflammatory condition rather than only a dermatological disease.

The development of psoriasis is predominantly driven by the interleukin-23 (IL-23)/T helper 17 (Th17) axis. In particular, IL-23 (released by activated myeloid dendritic cells) promotes the differentiation and proliferation of Th17 lymphocytes, leading to an increased production of pro-inflammatory cytokines, such as IL-22, IL-17A and IL-17F, causing keratinocytes proliferation and impairing their differentiation, thus contributing to the typical psoriatic lesions phenotype [8]. Moreover, the cytokines released by the hyperproliferative keratinocytes create positive feedback that perpetuates the inflammatory condition [8,9].

Importantly, the IL-23/Th17 pathway is also involved in the pathogenesis of several systemic inflammatory and metabolic disorders, suggesting a complex interaction between inflammation, metabolism, and nutritional balance in chronic inflammatory diseases [10,11,12]. Based on this evidence, the assessment of immune-nutritional status, associated with the tools commonly used to detect disease activity, such as the Psoriasis Area and Severity Index (PASI) and the DLQI (Dermatology Life Quality Index), has emerged as a valuable tool for capturing this complex interaction. Further common parameters that reflect immune activation and disease burden are the C-reactive protein (CRP), erythrocyte sedimentation rate (ESR), and leukocyte and platelet counts [13,14,15]. Moreover, composite indices derived from routine laboratory parameters, such as the neutrophil-to-lymphocyte ratio (NLR), platelet-to-lymphocyte ratio (PLR), lymphocyte-to-monocyte ratio (LMR) and systemic immune-inflammation index (SII), associated with the hemoglobin–albumin–lymphocyte–platelet (HALP) score, Controlling Nutritional Status (CONUT) score, Prognostic Nutritional Index (PNI), neutrophile-to-albumin ratio (NAR) and monocyte-to-albumin ratio (MAR), have gained attention as integrative markers of immune activation and nutritional status [16,17,18,19]. Another indicator of nutritional status and inflammatory activity is serum albumin. Hypoalbuminemia may reflect chronic inflammation and impaired nutritional balance [20,21]. The metabolic and cardiometabolic markers commonly altered in psoriasis, such as fasting glucose, lipid profile (total cholesterol, LDL, HDL, and triglycerides), triglycerides-to-HDL cholesterol ratio (TG/HDL), LDL/HDL ratio and total cholesterol/HDL ratio, provide additional insight into metabolic dysfunction and systemic inflammation [22,23,24].

The introduction of targeted biologic therapies that interact with specific cytokines rather than induce general immunosuppression have resulted in safer and more effective treatments against psoriasis compared to previous medications [25]. Among these, Tildrakizumab, a selective monoclonal antibody targeting IL-23, and Bimekizumab, a monoclonal antibody that recognizes a domain shared by both IL-17A and IL-17F, provide effective and sustained suppression of the key pathogenic pathways responsible for psoriasis onset and progression [26,27,28,29,30,31]. However, their impact on systemic inflammation and immune-nutrition parameters is still not understood completely.

Based on all these findings, the aim of the present exploratory study was to evaluate the immune-nutrition status of patients with plaque psoriasis treated with Tildrakizumab or Bimekizumab, by assessing changes in their biochemical and metabolic parameters before and after biologic therapy with each treatment independently, and exploring the potential associations with clinical outcomes. The goal was not a direct comparison between treatments.

## 2. Results

### 2.1. Demographic Data

All the demographic and clinical characteristics of the patients enrolled in the present study are summarized in Table 1. The Bimekizumab group was mainly composed of male subjects (19 males, nine females). Of these, 15 patients had started treatment before September 2024 and were retrospectively assessed, whereas three patients started treatment in September 2024 and were prospectively followed. The mean age was 48.67 ± 15.34 years. The mean age at psoriasis onset was 31.29 ± 15.41 years. Concerning the anthropometric parameters, there was a non-significant trend of increase in body weight between baseline (Time 0) and week 52 (84.73 ± 18.66 kg and 85.88 ± 12.35 kg, respectively). Similarly, the Body Mass Index (BMI) was not significantly increased at week 52 compared to baseline (28.52 ± 5.64 and 28.84 ± 3.55, respectively). The Bio-experience was assessed at 36%.

The Tildrakizumab group was composed of an equal male and female composition (13 males and 11 females). Of these, 22 patients had started treatment before September 2024 and were retrospectively assessed, whereas two patients started treatment in September 2024 and were prospectively followed. The mean age was 55.76 ± 15.91. The mean age at psoriasis onset was 36.04 ± 17.33. The mean body weight at baseline (Time 0) did not differ significantly from that observed at week 52 (79.46 ± 14.10 and 80.82 ± 15.44 kg, respectively). Similarly, we reported comparable BMI values between baseline and week 52 (26.50 ± 3.75 and 26.67 ± 5.31). The Bio-experience was assessed at 56%.

Comorbidities were also reported for both groups, as shown in Figure 1. The Bimekizumab group’s comorbidities were as follows: 4% with tumors, 7% with metabolic disorders, 9% with type 2 diabetes mellitus (T2DM), 24% with dyslipidemia, 26% with CVD and 30% with others. The Tildrakizumab group was characterized by: 14% with tumors, 7% with metabolic disorders, 10% with T2DM, 14% with dyslipidemia, 24% with CVD and 31% with others.

We evaluated different clinical indexes, which showed that the cohort presented with moderate-to-severe disease at baseline (Table 2).

The mean PASI score was 13.32 ± 10.76 for the Bimekizumab and 14.79 ± 13.30 for the Tildrakizumab groups. Both treatments resulted in a significant reduction in the PASI score at week 52 (0.00 ± 0.00 for Bimekizumab, *p* < 0.0001; 2.35 ± 3.53 for Tildrakizumab, *p* < 0.001) (Figure 2a,b). The assessed disease severity had a moderate impact on quality of life, as reflected by mean DLQI scores of 7.52 ± 8.31 for the Bimekizumab and 9.88 ± 3.98 for the Tildrakizumab groups. Only the Bimekizumab group reached a significantly improved DLQI at week 52 (0.00 ± 0.00, *p* < 0.001) (Figure 2c,d).

### 2.2. Glucose and Lipid Profile

In Table 3**,** the biochemical assessment performed on the study population at baseline and at week 52 for both treatments are reported. No statistically significant differences in glucose metabolism were observed in the two groups. The total cholesterol levels showed a non-significant trend of increase in the Bimekizumab group, while a non-significant reduction was reported for the Tildrakizumab group. A trend of increase (although not significant) between baseline and week 52 was reported for the LDL levels for both groups. A slight trend of increase was observed for the HDL levels in both treatment groups. A non-significant increase in the triglyceride (TG) levels was detected in the Bimekizumab group between baseline and week 52, while, in contrast, a slight trend of reduction in TG levels was reported in the Tildrakizumab group between baseline and week 52.

With regards to the lipid profile, we also assessed the cardiovascular disease (CVD) risk by evaluating the lipoproteins-to-cholesterol ratios. We observed a very slight increase in the TC/HDL ratio in the Bimekizumab group. Considering the LDL/HDL ratio, a slight trend of increase was observed in both groups between baseline and week 52. However, as observed in the Bimekizumab group, a slight increase in TG levels and in the TG/HDL ratio was reported between baseline and week 52, although the ratio remained classified in the low-risk category. A slight decrease was also reported in the Tildrakizumab group at the same time points. The non-high-density lipoprotein cholesterol to high-density lipoprotein cholesterol ratio (NHHR) was slightly, though not significantly, higher in the Bimekizumab group at week 52 compared to baseline. In contrast, the Tildrakizumab group showed a trend of reduction in this ratio, although the difference between baseline and week 52 did not reach a significant level.

### 2.3. Immunological and Inflammatory Profiles

The immunological and inflammatory profiles were also examined (Table 4). The Bimekizumab group showed a slight increase in neutrophile and monocyte levels between baseline and week 52. Conversely, the Tildrakizumab group showed a slight decreasing trend in both neutrophile and monocyte levels at week 52 compared to baseline. No significant differences were observed in the lymphocyte levels in either group. The platelet levels showed a non-significant trend of increase in both treatment groups.

A slight decrease was observed in the neutrophil-to-lymphocyte ratio (NLR) in both groups. However, a trend of decrease was reported for the platelet-to-lymphocyte ratio (PLR) at week 52 compared to baseline in the Bimekizumab group, while a slight increase was detected in the Tildrakizumab group. In contrast, a slight increase was reported for the lymphocyte-to-monocyte (LMR) ratio in both groups between baseline and week 52.

A significant reduction in the C-reactive protein (CRP) levels was observed in both groups between baseline and week 52 (2.43 ± 2.84 vs. 0.89 ± 1.12 for the Bimekizumab group, *p* < 0.05; 4.32 ± 6.31 vs. 2.50 ± 3.43 for the Tildrakizumab group, *p* < 0.05). The effect size magnitude indicated a strong effect in both the Bimekizumab and Tildrakizumab groups, further confirming the statistically significant reduction in CRP values. No significant changes were observed in the erythrocyte sedimentation rate (ESR) levels in either group. A slight trend of reduction was observed in the systemic immune-inflammation index (SII) in both groups between baseline and week 52.

### 2.4. Immuno-Nutritional Status Assessment

The immune-nutritional profile was assessed by evaluating well-established immune-nutritional markers in the study population, as described in the Materials and Methods Section (Table 5 and Figure 3).

First, we analyzed the albumin and hemoglobin levels. No significant differences were observed in either group. We then examined the hemoglobin, albumin, lymphocyte, platelet (HALP) score. A significant increase in the HALP score was reported in the Bimekizumab group between baseline and week 52 (49.43 ± 27.51 vs. 60.38 ± 17.48, respectively; *p* < 0.05) (Figure 3a). The Tildrakizumab group did not show significant differences in this parameter between baseline and week 52, although a trend of increase can be observed (Figure 3b). The effect size magnitude indicated a strong effect for Bimekizumab (confirming the significant *p* value) but not for Tildrakizumab, confirming the non-statistical relevance of the HALP increase in this study group. The Controlling Nutritional Status (CONUT) score, a screening tool for malnutrition risk, was significantly reduced at week 52 compared to baseline in the Bimekizumab (1.95 ± 1.05 vs. 0.76 ± 0.70, respectively; *p* < 0.0001) (Figure 3c) group, while a slight decrease was observed in the Tildrakizumab group (Figure 3d). The effect size magnitude indicated a strong effect for Bimekizumab (confirming the significant *p* value) but not for Tildrakizumab, confirming the non-statistical relevance of the CONUT variation in this study group. No significant alterations were observed in the Prognostic Nutritional Index (PNI) in either group. Additional indices reflecting the interplay between immune and nutritional status included the neutrophile-to-albumin ratio (NAR) and the monocyte-to-albumin ratio (MAR). No significant changes were reported for the Bimekizumab group; a slight reduction in the NAR was observed in the Tildrakizumab group between baseline and week 52. No significant changes were detected for the MAR in either treatment group.

## 3. Discussion

The biologic therapies currently approved for the treatment of psoriasis are specifically designed for patients affected by chronic moderate-to-severe disease, often long-standing and refractory to conventional systemic disease-modifying anti-rheumatic drugs (DMARDs) [1]. Psoriasis is now widely recognized as a systemic inflammatory disorder, frequently associated with metabolic comorbidities, such as hypercholesterolemia, type 2 diabetes mellitus, and metabolic syndrome [2,3]. Given that these agents are intended for long-term administration, in the context of a disease characterized by chronicity and therapeutic resistance, increasing attention must be paid to their role within the broader immuno-metabolic framework of affected patients [5,6]. In this regard, the immuno-nutritional evaluation of psoriatic patients undergoing biologic therapy represents an integrated approach aimed at assessing the interplay between nutritional status, systemic inflammation, and therapeutic response, including both prognostic and therapeutic implications [6,8,9,10].

Subcutaneous adipose tissue located under psoriatic skin showed a strong pro-inflammatory transcriptional profile, with enrichment of innate immune and inflammatory pathways. Signals related to keratinization and epidermal differentiation were likely due to dermal contamination in full-thickness biopsies rather than adipocyte-specific changes [11].

Psoriasis is a prototypical immune-mediated disease characterized by chronic systemic inflammation extending well beyond cutaneous manifestations [12]. The nutritional status directly influences disease activity, largely through the pro-inflammatory effects of adipose tissue and its capacity to modulate immune responses [13,14]. This intricate interconnection between metabolism, immunity, and inflammation constitutes the conceptual basis of the immuno-nutritional approach. Within this context, several systemic inflammatory indices derived from routine hematological parameters have emerged as practical, cost-effective, and readily accessible tools for evaluating systemic inflammation and monitoring responses to biologic therapies in psoriatic patients [14].

In our cohort, both biologic agents demonstrated excellent therapeutic efficacy in terms of clinical improvement, as assessed by the PASI. Notably, the patients treated with Bimekizumab achieved complete skin clearance (PASI 0) at week 52, whereas those receiving Tildrakizumab reached a mean PASI slightly above two at the same time point. Although the baseline DLQI scores did not indicate a markedly impaired quality of life, both treatment groups exhibited complete normalization of DLQI at week 52, reflecting the well-established correlation between clinical skin improvement and reduction in disease burden on daily functioning. These outcomes were accompanied by an excellent safety profile for both drugs, albeit with some differences in their interaction with immuno-nutritional parameters.

Psoriatic patients are known to exhibit increased fasting glucose levels and a higher risk of developing type 2 diabetes mellitus compared to the general population, with a dose-dependent relationship linked to disease severity [15,16]. Indeed, psoriasis severity correlates directly with circulating glucose levels, and the risk of diabetes increases proportionally with the extent of body surface area involved [18]. Hyperglycemia in psoriasis is largely mediated by the IL-17 axis, which impairs insulin secretion without inducing significant pancreatic islet inflammation. Additionally, TNF-α is also secreted by adipocytes, inhibits insulin receptor activity and reduces the expression of the glucose transporter GLUT4, thereby promoting insulin resistance [17,19]. TNF-α promotes elevated circulating levels of free fatty acids and triglycerides, thereby contributing to the development of atherogenic dyslipidemia [21].

IL-23 inhibitors have been shown to significantly reduce fasting glucose and insulin levels after three months of treatment, with effects comparable to IL-17 inhibitors; however, some evidence suggests superior long-term metabolic efficacy of IL-23 blockade [20,22,23]. Psoriatic patients also display altered adipokine profiles, characterized by increased leptin and resistin and decreased adiponectin, further contributing to insulin resistance [24]. In our cohort, no statistically significant differences in fasting glucose levels were observed between baseline and week 52 in either treatment group, although larger sample sizes may be required to detect subtle metabolic changes over time.

Lipid metabolism is also heavily altered in psoriasis, with patients typically exhibiting elevated LDL cholesterol levels and a predominance of small, dense LDL particles, a profile resembling the one observed in diabetic patients. A randomized controlled trial demonstrated that three months of anti-IL-17 biologic therapy does not significantly modify LDL-C levels [22]. However, a recent meta-analysis (2026) reported that the long-term use of IL-17A inhibitors is associated with a significant increase in triglyceride levels [25]. The triglyceride-to-HDL ratio (TG/HDL) represents a surrogate marker of insulin resistance and atherogenic dyslipidemia, characterized by small, dense LDL particles [27]. This ratio is particularly relevant in psoriasis, given the increased cardiovascular risk, subclinical atherosclerosis, and vascular inflammation associated with the disease [2]. Dyslipidemia is highly prevalent in psoriatic patients and much more probable compared to the general population. Current AAD-NPF guidelines recommend periodic lipid screening in patients with moderate-to-severe psoriasis [26]. In our cohort, the increase in triglyceride levels observed among the Bimekizumab-treated patients was accompanied by a statistically significant rise in the TG/HDL ratio, whereas the patients receiving Tildrakizumab exhibited only a mild, non-statistically significant upward trend. This finding may be explained by the increase in absolute triglyceride concentrations in the Bimekizumab group, potentially resulting from IL-17 pathway inhibition, which may have impaired the downregulation of PPAR-γ expression and reduced activation of the WNT signaling pathway, ultimately promoting the expression of genes involved in lipid synthesis and accumulation [28,29].

The non-HDL cholesterol to HDL ratio (NHHR) has emerged as a superior marker compared to LDL-C for the assessment of subclinical atherosclerosis and carotid plaque vulnerability. Due to the systemic inflammatory burden, psoriatic patients frequently exhibit elevated NHHR values, reflecting an atherogenic lipid profile characterized by increased VLDL and LDL and reduced HDL levels [31]. The NHHR has been validated as a robust predictor of cardiovascular risk and mortality, and its monitoring during biologic therapy provides an integrated parameter reflecting vascular inflammation beyond PASI improvement [30]. In our study, non-statistically significant modulation of NHHR was reported, probably due to the limited sample size.

The neutrophil-to-lymphocyte ratio (NLR), reflecting the balance between innate and adaptive immunity, is a well-established marker of systemic inflammation [32]. Psoriatic patients exhibit significantly higher NLR values compared to healthy controls, with higher values observed in moderate-to-severe cases [33]. An elevated baseline-derived NLR is an independent predictor of early super-responder status, while the NLR is also the strongest predictor of psoriatic arthritis among psoriatic patients [34,35]. Although the NLR typically decreases after 3–4 months of biologic therapy and correlates with PASI improvement, in our cohort we did not observe statistically significant changes, despite a mild decreasing trend in both treatment groups.

Similarly, the lymphocyte-to-monocyte ratio (LMR), a sensitive indicator of the balance between adaptive immunity and innate monocytic activation, reflects systemic immune activation in psoriasis [36]. Low LMR values are associated with increased carotid intima-media thickness and arterial stiffness, markers of subclinical atherosclerosis. Conversely, an increase in the LMR during treatment with anti-IL-17 or anti-IL-23 agents suggests not only disease control but also a potential reduction in cardiovascular risk [37,38]. In our cohort, a trend of increase in the LMR was observed in both groups; the increase did not reach a significant level, probably due to the small sample size.

The PLR integrates information on platelet reactivity and adaptive immune reserve, a valuable hematological marker in dermatological practice [39,40]. Although its accuracy related to the PASI remains debated, the PLR is consistently elevated in psoriatic patients compared to healthy controls, reflecting a systemic inflammatory burden [41]. In this regard, we documented, in both the Bimekizumab- and Tildrakizumab-treated groups, a decreasing (non-significant) trend in the PLR at week 52.

The C-reactive protein, an acute-phase reactant primarily induced by IL-6, serves as a key link between cutaneous and systemic inflammation in psoriasis [42]. A significant linear correlation exists between CRP levels and PASI scores; moreover, an elevated CRP is an independent predictor of cardiovascular events, with levels persistently >3 mg/L indicating high vascular risk due to endothelial inflammation [36,43]. In our study, the CRP levels were significantly reduced in both treatment groups, consistent with the reduction in systemic inflammatory burden accompanying clinical improvement.

The systemic immune-inflammation index (SII), which integrates neutrophils, lymphocytes, and platelets, provides a comprehensive overview of innate immune activation, adaptive response, and pro-thrombotic platelet activity [44,45]. Elevated SII values are associated with active psoriasis and correlate positively with the PASI, although some studies suggest that the SII may be more useful as a marker of systemic risk rather than disease extent [46,47]. The SII is also a robust marker for identifying patients at risk of metabolic syndrome, hepatic steatosis, hypertension, and coronary artery disease [48,49]. In our cohorts, a non-significant decreasing trend in the SII was observed. Näslund-Koch C et al. found, among 105 418 individuals, that low-grade systemic inflammation, as measured by the SII, NLR and CRP, was an independent risk factor for psoriasis, especially in moderate-to-severe cases. These findings support the hypothesis that low-grade systemic inflammation may contribute to the pathogenesis of psoriasis rather than simply be a consequence of the disease [32].

Among the composite immuno-nutritional indices, the HALP score uniquely integrates nutritional and immunological parameters. Unlike other indices that increase with inflammation, HALP values are inversely associated with disease risk and the systemic inflammatory burden. Low HALP values reflect a state of increased inflammation and impaired nutritional status, both commonly observed in psoriasis [50,51]. To date, Min Fan et al. are the only authors to have demonstrated, using a national dataset, an association between higher HALP values and an increased risk of developing psoriasis. In their study, elevated HALP levels were significantly associated with a greater likelihood of developing psoriasis [50]. In our study, we observed a statistically significant increase in HALP values in the Bimekizumab-treated cohort, along with a non-significant increasing trend in patients who received Tildrakizumab. These findings suggest a potential improvement in the overall immuno-nutritional status, particularly in patients treated with IL-17 inhibition.

Consistently, the CONUT score, which reflects nutritional impairment and immune competence [52], showed a statistically significant reduction in both treatment groups. This decrease indicates an improvement in nutritional and immunological balance, further supporting the hypothesis that effective biologic therapy may positively modulate the immune-metabolic profile of psoriatic patients [20,53].

Overall, obesity and metabolic dysfunction are associated with dysregulated innate immune responses and increased neutrophilic activity, contributing to early resistance to biologic therapies, particularly those targeting the IL-17 and IL-23 pathways, which act mainly on adaptive immune responses. The findings of this study suggest that biological therapies may facilitate a systemic realignment, shifting the physiological profile from chronic inflammatory stress to metabolic and nutritional stability. The synchronized reduction in the CRP, triglycerides, and the TG/HDL ratio, combined with the improved HALP and CONUT scores, indicate a clinical reversal of the “psoriatic march” [54].

Within this framework, the integration of immuno-nutritional indices into clinical practice may provide a more comprehensive understanding of treatment response, extending beyond skin clearance to encompass systemic inflammation and metabolic health. Figure 4 shows a graphical representation summarizing the relevant role of the immune-nutritional assessment using the HALP score for predicting the outcomes among psoriatic patients.

However, some limitations should be acknowledged. The study was exploratory and at a single center with a small sample size. Moreover, the treatment allocation was not randomly assigned, but based on each patient’s diagnosis, leading to a possible baseline imbalance. Although the large number of endpoints analyzed may have increased the possibility of a Type I error, a multiple statistical comparison was not applied due to the exploratory nature of the study. The use of retrospective data collection for some participants also created some limitations, including incomplete data and possible information and selection bias. The absence of a direct nutritional assessment represents a limitation of the present study. We only provide an indirect evaluation of the nutritional status by using the routinely available laboratory parameters, which cannot fully replace a nutritional evaluation.

## 4. Materials and Methods

### 4.1. Study Population

In this observational ambispective clinical study, we enrolled 52 patients affected by moderate-to-severe psoriasis, who were recruited at the Dermatology Unit of Tor Vergata University Hospital in Rome. The study started in September 2024, and follow-up continued until September 2025. Due to the exploratory nature of the study, the sample size was assessed with a convenience/available-patient sample; thus, the sample size was determined by the number of eligible patients meeting the inclusion criteria during the study period. All patients enrolled in this study signed an informed consent.

The choice of biologic therapy was primarily guided by the pharmacoeconomic recommendations issued by the Lazio Regional Health Authority. Additional clinical factors were considered when selecting the specific biologic agent, based on routine clinical practice and individual patient characteristics. In particular, a history of recurrent fungal infections, uncontrolled diabetes mellitus, or inflammatory bowel disease favored the use of Tildrakizumab, whereas a higher baseline PASI score, concomitant psoriatic arthritis, and a younger age favored the prescription of Bimekizumab.

Twenty-eight patients were treated with Bimekizumab (UCB, Bruxelles, Belgium) at a dose of 320 mg every 4 weeks for 16 weeks, followed by a maintenance regimen of 320 mg every 8 weeks, while twenty-four patients received 100 mg of Tildrakizumab (Almirall, Barcellona, Spain) at baseline, at week 4, and every 12 weeks thereafter.

The inclusion criteria were: age >18 years; patients with a diagnosis of plaque psoriasis; and patients eligible for treatment with Bimekizumab or Tildrakizumab based on both the skin and comorbidities burden. The exclusion criteria were: age < 18 years at the time of enrollment; pregnant or breastfeeding patients; and patients with contraindications to the use of Bimekizumab or Tildrakizumab. In the entire group of patients, we observed no adverse effects to either treatment, nor any need for interruption of therapy.

Each patient underwent a baseline screening visit (T0), which determined the severity of psoriasis by assessment of clinic scores, such as the PASI (Psoriasis Area and Severity Index) and DLQI (Dermatology Life Quality Index). Demographic data were also collected, including year of disease onset and its duration, family history of psoriasis, previous drug treatments, smoking, body weight, and BMI (Body Mass Index). Patients were also screened for the presence of associated comorbidities, including type 2 diabetes, metabolic disease, dyslipidemia, cardiovascular disease, tumors and others.

This study was conducted in accordance with the ICH/GCP guidelines and in compliance with the Declaration of Helsinki. The experimental procedure was approved by the Institutional Review Board’s (IRB) Independent Ethical Committee of “Tor Vergata” University Hospital (R.S. 139.23, approved on 26th of May, 2023).

### 4.2. Clinical and Biochemical Assessment

At baseline and at week 52, we assessed: the glucose and lipid profile (glycemia, total cholesterol, LDL, HDL, and triglycerides), cardiovascular risk (total cholesterol/HDL, LDL/HDL, triglycerides/HDL, and non-high-density lipoprotein cholesterol to high-density lipoprotein cholesterol ratio (NHHR)), immunological and inflammatory profile (neutrophile, lymphocyte, platelet, monocyte, CRP, ESR, NLR, PLR, LMR and SII score) and immuno-nutritional indices (HALP score, CONUT score, PNI, NAR and MAR).

The calculation methods for the immune-nutritional indicators were previously assessed by Jia et al. [55].

- HALP score: [Hemoglobin (g/L) × albumin (g/L) × lymphocytes (×10^9^)]/platelets (×10^9^);
- PNI: Albumin (g/L) + 5×lymphocyte count (×10^9^);
- NAR: Neutrophil count (×10^9^)/albumin (g/ L);
- MAR: Monocyte count (×10^9^)/albumin (g/ L);
- CONUT score: Albumin score (g/dL) + TLC (Total lymphocyte) score + T-cho score.

The albumin score was assessed as follows: 1 (3.5–4.5) or 2 (30–34.9), The TLC score was 0 (≥1600), 1 (1200–1599), 2 (800–119) or 3 (800). The total cholesterol score was assessed as follows: 0 (≥180), 1 (140–180), 2 (100–139) or 3 (100).

For patients who had already started therapy at the time of enrollment, their baseline values and follow-up data were extracted retrospectively from their medical records, ensuring the availability of reliable information on the biochemical and clinical markers at the time points required by the study.

### 4.3. Statistical Analysis

The statistical analysis was conducted using the GraphPad Prism 9 program (La Jolla, CA, USA). The assumption of normality was assessed by performing a Kolmogorov–Smirnov test. After that, a paired analysis was performed. In particular, a paired *t* test was used for normally distributed samples, while a Wilcoxon test was used for non-normally distributed samples. The effect size was also calculated by evaluating the Rank-Biserial Correlation based on the data given by GraphPad (sum of signed ranks, *W*) and the following online calculation:

$$S_{max}=\frac{N(N+1)}{2}\;\mathrm{and}\;r_{rb}=\frac{W}{S_{max}}$$

where *N* is the number of pairs considered. An absolute *r_rb_* value of 0.10 indicates a small size effect, a value of 0.30 indicates a medium effect, and values equal to or higher than 0.50 indicate a strong size effect.

Values for which the probability was less than 5% (*p* < 0.05) were considered statistically significant.

## 5. Conclusions

In this preliminary descriptive real-world study, both Bimekizumab and Tildrakizumab demonstrated clinical efficacy by reducing the systemic inflammatory burden in our study population. Moreover, our findings support a preliminary association among biologic treatments; general clinical improvement; and indirect laboratory evaluation of immune-nutritional status, particularly the HALP and CONUT scores. This highlights the potential value of integrating inflammatory and immune-nutritional biomarkers (associated with a complete nutritional evaluation) for the assessment of patients with psoriasis, to better personalize therapeutic strategies and monitor the systemic disease burden. However, larger prospective and adequately powered studies are required to confirm the clinical and pharmacological relevance.

## Figures and Tables

**Figure 1:**
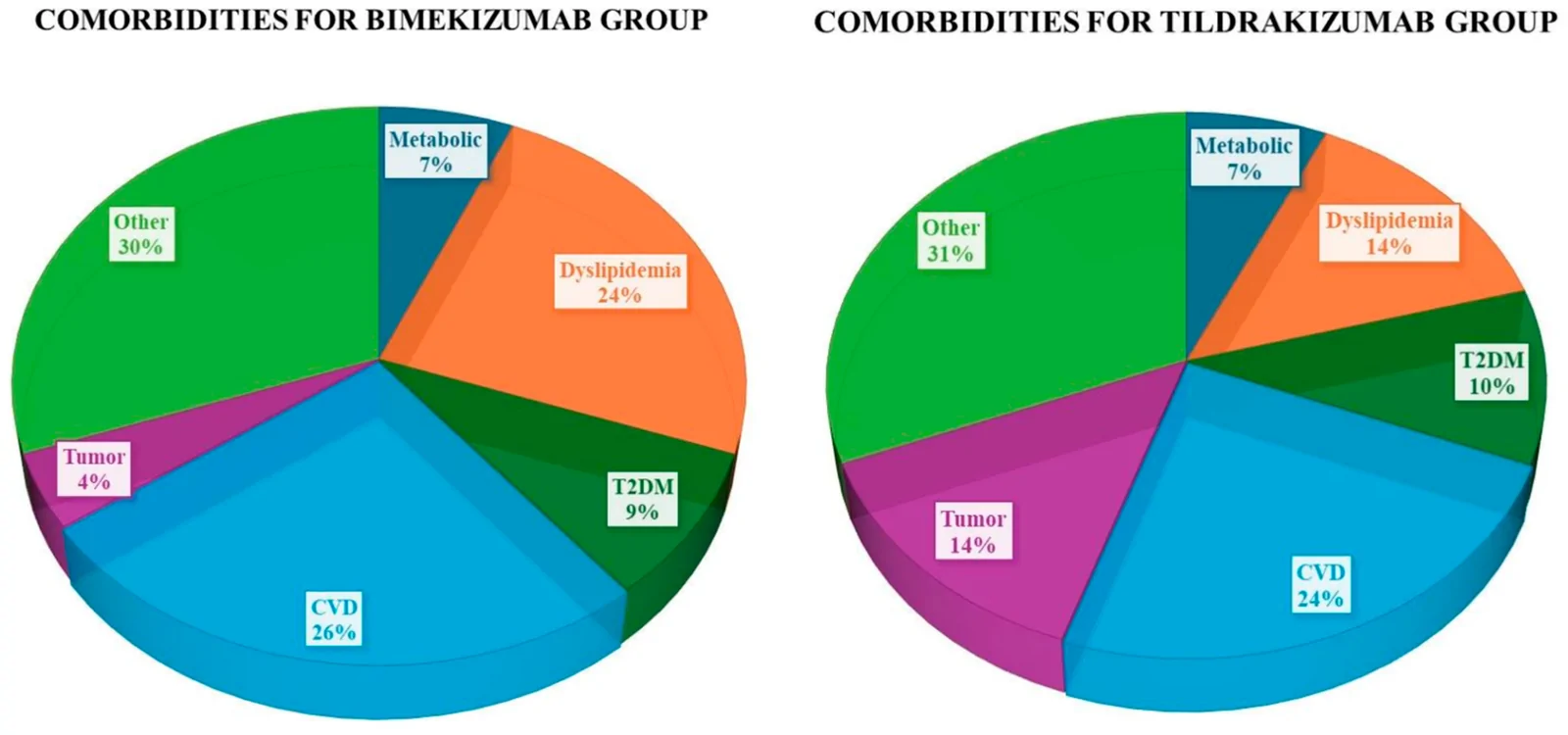
Overall distribution of comorbidities for both the Bimekizumab and Tildrakizumab groups. The percentages relate to the number of comorbidities in the study groups, which provide their overall prevalence. CVD: cardiovascular disease; T2DM: Type 2 Diabetes Mellitus.

**Figure 2:**
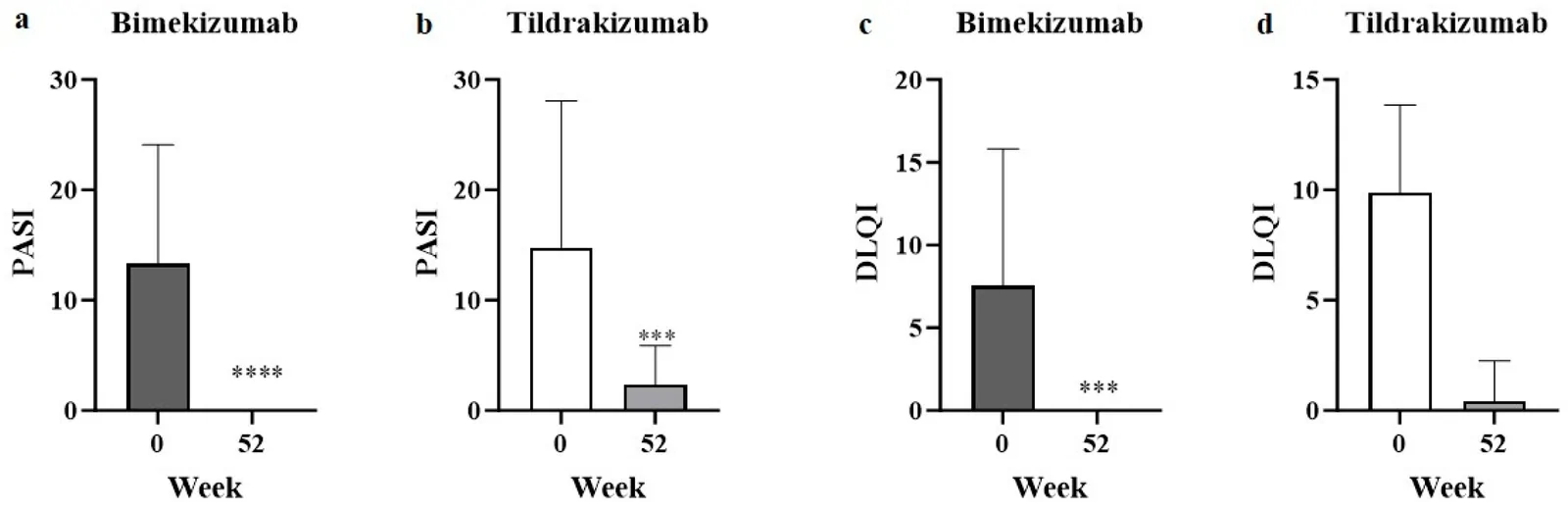
Clinical index analysis. (**a**,**b**) PASI and (**c**,**d**) DLQI scores were analyzed in the study population at baseline and at week 52. *** *p* < 0.001; **** *p* < 0.0001. PASI: Psoriasis Area and Severity Index; DLQI: Dermatology Life Quality Index.

**Figure 3:**
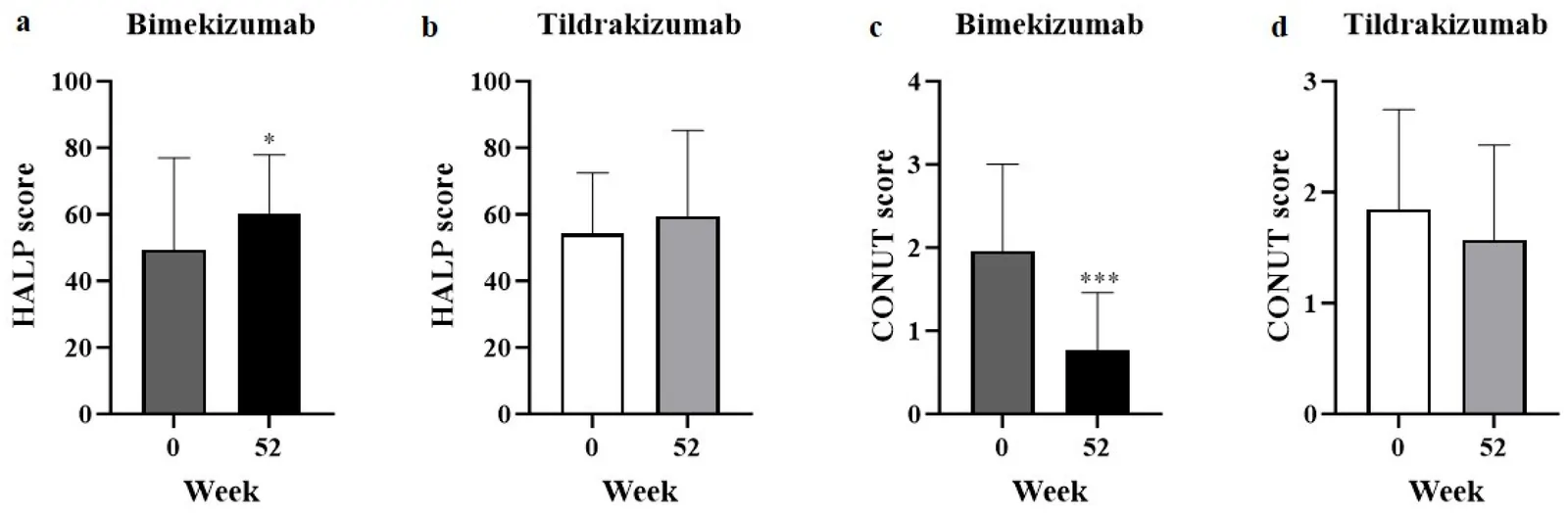
Immuno-nutritional assessment. (**a**,**b**) HALP scores for the Bimekizumab and Tildrakizumab groups, respectively, and (**c**,**d**) CONUT scores for the Bimekizumab and Tildrakizumab groups, respectively, at baseline and at week 52. * *p* < 0.05, *** *p* < 0.001. HALP score: Hemoglobin–albumin–lymphocyte–platelet score; CONUT score: Controlling Nutritional Status score.

**Figure 4:**
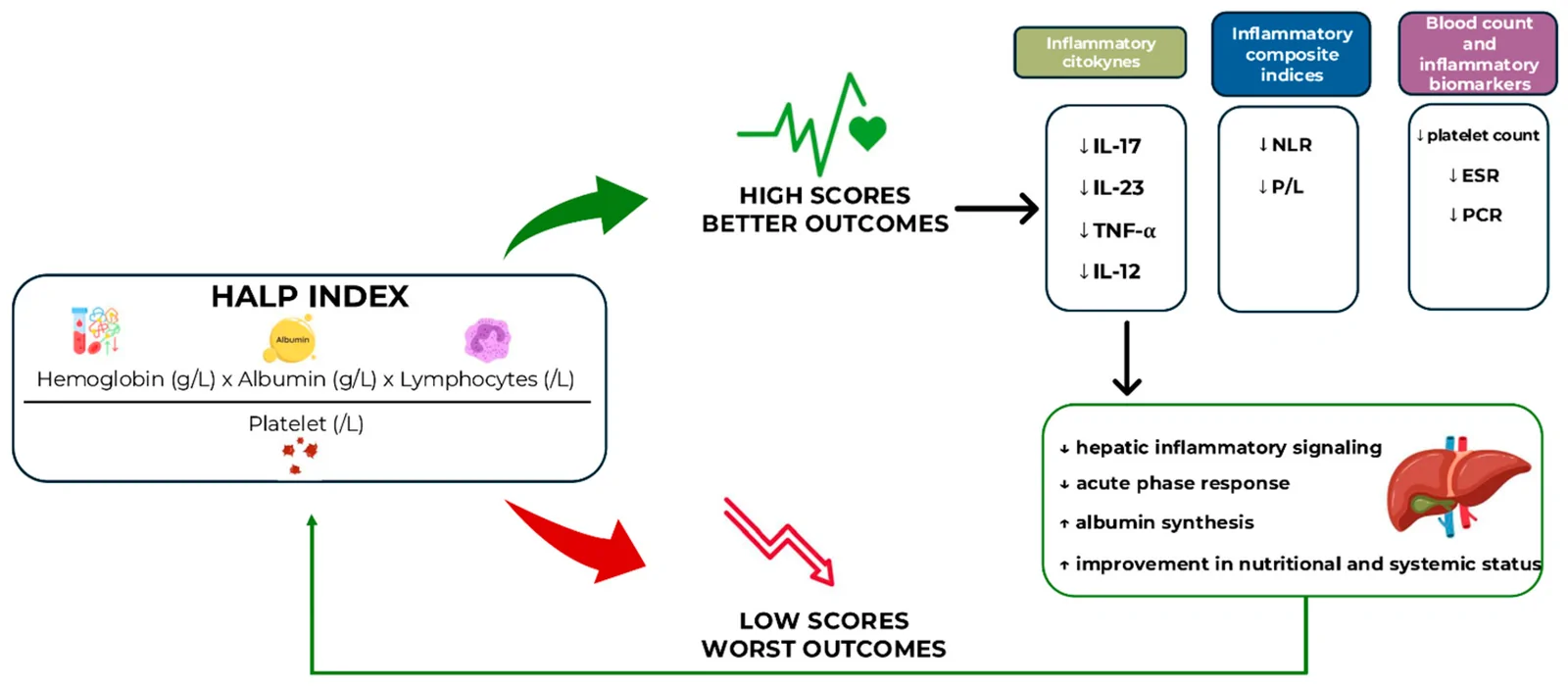
Graphical representation of the impact of HALP index on psoriatic patients’ outcomes.

**Table 1:** Demographic and clinical characteristics.

Demographic and Clinical Characteristics	Bimekizumab (N = 28)	Tildrakizumab (N = 24)
Sex Male, N (%) Female, N (%)	19 (67.8) 9 (32.2)	13 (54.2) 11 (45.8)
Mean Age (years)	48.67 ± 15.34	55.76 ± 15.91
Mean Age of Psoriasis Onset (years)	31.29 ± 15.41	36.04 ± 17.33
Mean Weight (kg) Baseline (T = 0) Week 52	84.73 ± 18.66 85.88 ± 12.35	79.46 ± 14.10 80.82 ± 15.44
Mean BMI (kg/m^2^) Baseline (T = 0) Week 52	28.52 ± 5.64 28.84 ± 3.55	26.50 ± 3.75 26.67 ± 5.31
Bio-experience	36%	56%

BMI: Body Mass Index.

**Table 2:** Clinical index assessment.

Clinical Index	Bimekizumab (N = 28)	Tildrakizumab (N = 24)
PASI Baseline (T = 0) Week 52	13.32 ± 10.76 0.00 ± 0.00 ****	14.79 ± 13.30 2.35 ± 3.53 ***
DLQI Baseline (T = 0) Week 52	7.52 ± 8.31 0.00 ± 0.00 ***	9.88 ± 3.98 0.42 ± 1.83

PASI: Psoriasis Area and Severity Index; DLQI (Dermatology Life Quality Index); *** *p* < 0.001; **** *p* < 0.0001.

**Table 3:** Glucose and lipid metabolic profiles.

Biochemical Parameters	Bimekizumab (N = 28)	Tildrakizumab (N = 24)
Glycemia (mg/dL) Baseline (T = 0) Week 52	92.90 ± 22.16 92.29 ± 10.86	94.68 ± 23.10 97.72 ± 25.99
Total Cholesterol (TC) (mg/dL) Baseline (T = 0) Week 52	166.90 ± 34.55 175.40 ± 35.41	187.90 ± 49.11 181.30 ± 49.06
LDL (mg/dL) Baseline (T = 0) Week 52	105.10 ± 38.18 106.50 ± 37.46	114.60 ± 48.60 120.40 ± 51.52
HDL (mg/dL) Baseline (T = 0) Week 52	48.56 ± 11.10 48.52 ± 14.91	53.80 ± 18.52 56.00 ± 14.44
Triglycerides (TG) (mg/dL) Baseline (T = 0) Week 52	92.11 ± 41.25 122.80 ± 41.85	116.60 ± 74.60 101.20 ± 59.58
TC/HDL Baseline (T = 0) Week 52	3.63 ± 1.10 3.93 ± 1.46	3.37 ± 0.95 3.35 ± 0.98
LDL/HDL Baseline (T = 0) Week 52	2.32 ± 1.06 2.40 ± 1.16	2.09 ± 0.78 2.11 ± 0.81
TG/HDL Baseline (T = 0) Week 52	1.85 ± 0.98 2.94 ± 1.42	2.60 ± 2.52 1.98 ± 1.95
NHHR Baseline (T = 0) Week 52	2.63 ± 1.10 2.93 ± 1.46	2.37 ± 0.95 2.35 ± 0.98

LDL: low-density lipoprotein; HDL: high-density lipoprotein; NHHR: non-high-density lipoprotein cholesterol to high-density lipoprotein cholesterol ratio.

**Table 4:** Immunological and inflammatory profiles assessment.

Immunological and Inflammatory Profiles	Bimekizumab (N = 28)	Tildrakizumab (N = 24)
Neutrophile (10^3^/µL) Baseline (T = 0) Week 52	3.96 ± 1.45 4.09 ± 1.46	4.55 ± 1.39 4.41 ± 1.97
Lymphocyte (10^3^/µL) Baseline (T = 0) Week 52	2.03 ± 0.74 2.17 ± 0.57	2.11 ± 0.54 2.14 ± 0.62
Platelets (10^3^/µL) Baseline (T = 0) Week 52	222.10 ± 52.38 225.50 ± 45.46	262.80 ± 65.08 264.80 ± 62.93
Monocyte (10^3^/µL) Baseline (T = 0) Week 52	0.49 ± 0.17 0.54 ± 0.22	0.52 ± 0.16 0.45 ± 0.14
NLR Baseline (T = 0) Week 52	2.39 ± 1.69 2.21 ± 0.99	2.28 ± 0.88 2.06 ± 0.89
PLR Baseline (T = 0) Week 52	128.60 ± 82.98 108.90 ± 25.98	130.70 ± 43.28 134.50 ± 51.62
LMR Baseline (T = 0) Week 52	4.39 ± 1.76 4.43 ± 1.64	4.14 ± 1.65 4.86 ± 1.99
CRP (mg/L) Baseline (T = 0) Week 52 Effect size	2.43 ± 2.84 0.89 ± 1.12 * 0.64	4.32 ± 6.31 2.50 ± 3.43 * 0.60
ESR (mm/h) Baseline (T = 0) Week 52	18.43 ± 15.16 15.50 ± 13.36	21.87 ± 20.21 10.36 ± 7.34
SII Baseline (T = 0) Week 52	516.30 ± 362.90 470.90 ± 206.70	538.00 ± 217.40 433.10 ± 188.20

NLR: neutrophil-to-lymphocyte ratio; PLR: platelet-to-lymphocyte ratio; LMR: lymphocyte-to-monocyte ratio; CRP: C-reactive protein; ESR: erythrocyte sedimentation rate; SII: systemic immune-inflammation index; * *p* < 0.05.

**Table 5:** Immuno-nutritional profile assessment.

Clinical Index	Bimekizumab (N = 28)	Tildrakizumab (N = 24)
Albumin (g/L) Baseline (T = 0) Week 52	43.10 ± 4.26 42.43 ± 2.94	43.01 ± 3.67 42.64 ± 2.09
Hemoglobin (g/L) Baseline (T = 0) Week 52	146.60 ± 15.44 147.50 ± 11.41	144.90 ± 11.87 148.80 ± 14.15
HALP score Baseline (T = 0) Week 52 Effect size	49.43 ± 27.51 60.38 ± 17.48 * 0.71	54.14 ± 18.31 59.37 ± 25.88 0.15
CONUT score Baseline (T = 0) Week 52 Effect size	1.95 ± 1.05 0.76 ± 0.70 *** 0.67	1.85 ± 0.90 1.57 ± 0.85 0.03
PNI Baseline (T = 0) Week 52	53.21 ± 6.46 51.78 ± 4.34	53.57 ± 3.97 53.05 ± 4.99
NAR Baseline (T = 0) Week 52	0.10 ± 0.04 0.10 ± 0.03	0.11 ± 0.04 0.09 ± 0.04
MAR Baseline (T = 0) Week 52	0.01 ± 0.004 0.01 ± 0.005	0.02 ± 0.03 0.01 ± 0.004

HALP score: hemoglobin–albumin–lymphocyte–platelet score; CONUT score: Controlling Nutritional Status score; PNI: Prognostic Nutritional Index; NAR: neutrophile-to-albumin ratio; MAR: monocyte-to-albumin ratio; * *p* < 0.05, *** *p* < 0.0001.

## Data Availability

The original contributions presented in this study are included in the article. Further inquiries can be directed to the corresponding author.

## Abbreviations

The following abbreviations are used in this manuscript:

BMI	Body Mass Index
CONUT score	Controlling Nutritional Status Score
CRP	C-Reactive Protein
CVD	Cardiovascular Disease
DLQI	Dermatology Life Quality Index
DMARDs	Disease-modifying Anti-Rheumatic Drugs
ESR	Erythrocyte Sedimentation Rate
HALP score	Hemoglobin, Albumin, Lymphocyte, and Platelet Score
HDL	High-Density Lipoprotein
LDL	Low-Density Lipoprotein
LMR	Lymphocyte-to-Monocyte Ratio
MAR	Monocyte-to-Albumin Ratio
NAR	Neutrophile-to-Albumin Ratio
NHHR	Non-High-Density Lipoprotein Cholesterol to High-Density Lipoprotein Cholesterol Ratio
NLR	Neutrophil-to-Lymphocyte Ratio
PASI	Psoriasis Area and Severity Index
PLR	Platelet-to-Lymphocyte Ratio
PNI	Prognostic Nutritional Index
SII	Systemic Immune-Inflammation Index
TC	Total Cholesterol
TG	Triglycerides
